# Prenatal Perfluoroalkyl Substance Exposure in Association with Global Histone Post-Translational Methylation in 2-Year-Old Children

**DOI:** 10.3390/toxics12120876

**Published:** 2024-11-30

**Authors:** Wan-Ju Tsai, Wu-Shiun Hsieh, Pau-Chung Chen, Chen-Yu Liu

**Affiliations:** 1 Institute of Environmental and Occupational Health Sciences, College of Public Health, National Taiwan University, Taipei 100, Taiwanpchen@ntu.edu.tw (P.-C.C.); 2 Department of Pediatrics, Cathay General Hospital, Taipei 106, Taiwan; hsiehws@ntu.edu.tw; 3 Department of Pediatrics, National Taiwan University College of Medicine, Taipei 100, Taiwan; 4 School of Medicine, College of Medicine, Kaohsiung Medical University, Kaohsiung 807, Taiwan; 5 Department of Environmental and Occupational Medicine, National Taiwan University Hospital and National Taiwan University College of Medicine, Taipei 100, Taiwan; 6 National Institute of Environmental Health Sciences, National Health Research Institutes, Miaoli 350, Taiwan; 7 Department of Public Health, College of Public Health, National Taiwan University, Taipei 100, Taiwan; 8 Global Health Program, College of Public Health, National Taiwan University, Taipei 100, Taiwan

**Keywords:** birth cohort study, perfluoroalkyl substances, prenatal exposure, epigenetics, histone post-translational modifications

## Abstract

Perfluoroalkyl substances (PFASs) have elimination half-lives in years in humans and are persistent in the environment. PFASs can cross the placenta and impact fetal development. Exposure to PFASs may lead to adverse effects through epigenetic mechanisms. This study aimed to investigate whether prenatal exposure to perfluorooctyl sulfonate (PFOS), perfluorooctanoic acid (PFOA), perfluorononanoic acid (PFNA), and perfluoroundecanoic acid (PFUA) was associated with global histone methylation level changes among the 130 2-year-old children followed-up in a birth cohort study in Taiwan. PFOS, PFOA, PFNA, and PFUA were measured by UHPLC/MS/MS in cord blood. Global histone methylation levels were measured from the blood leukocytes of 2-year-old children by Western blotting. Multivariable regression analyses were applied to adjust for potential confounding effects. Among the 2-year-old children, an IQR increase in the natural log-transformed PFUA exposure was associated with an increased H3K4me3 level by 2.76-fold (95%CI = (0.79, 4.73), *p* = 0.007). PFOA and PFNA exposures was associated with a decreased H3K27me3 level by 2.35-fold (95%CI = (−4.29, −0.41), *p* = 0.01) and 2.01-fold (95%CI = (−4.00, −0.03), *p* = 0.04), respectively. Our findings suggest that prenatal PFAS exposure affected histone post-translational modifications.

## 1. Introduction

Perfluoroalkyl substances (PFASs) are man-made compounds that consist of carbon chains with different chain lengths, in which the hydrogen atoms are substituted by fluorine atoms. PFASs have been manufactured since the 1950s and are widely used in daily and industrial products, such as food wrappers and non-stick cookware due to their water-, oil-, heat-, and stain-resistant properties [1]. The strong carbon–fluorine bond of PFASs makes PFASs persistent in the environment [2,3,4] and prevalent in many species, ranging from wildlife to plants and humans [5,6,7]. The average elimination half-lives of the two most widely studied PFASs, perfluorooctyl sulfonate (PFOS; C8) and perfluorooctanoic acid (PFOA; C8), in human serum are 5.4 years and 3.8 years, respectively [8]. PFASs can pass through the placenta [9] and have raised health concerns regarding prenatal exposure. Prenatal exposure to PFOS and PFOA may increase the risk of adverse effects at birth [10,11,12,13] and later in life, including endocrine-disrupting effects [14,15], immunotoxic responses [16], developmental or behavioral concerns [17,18], and cancer by a non-genotoxic mechanism [19]. PFOS and PFOA are considered non-genotoxic [20,21]. Many of the non-genotoxic xenobiotics have been shown to mediate gene expression and induce toxic effects by changing epigenetic regulations [22,23,24,25]. The PFOS- and PFOA-related adverse effects may be induced through epigenetic regulations.

Epigenetic regulation includes DNA methylation, histone modification, and microRNAs [26]. Histones are globular proteins including the histone fold regions and the less structured histone tails. Specific amino acids within these histone tails are targeted for post-translational modifications. These post-translational modifications, including acetylation, methylation, phosphorylation, and ubiquitination [27], may modulate the DNA damage response [28] and affect genomic stability and chromatin packaging by altering their interactions with the DNA and other chromatin-associated proteins [29]. The methyl modifications of histone are believed to turn over more slowly than the other modifications and are relatively more stable [30,31,32]. The sites of histone H3 modifications that are most widely studied are lysine 4, lysine 9, and lysine 27. The methylation of histones may be associated with gene activation or inactivation depending on the specific residue methylated. The trimethylation of lysine 4 of H3 (H3K4me3) has been linked to an open chromatin configuration, which is association with transcriptionally active promoters as well as less stability. In contrast, the trimethylation products of lysine 9 and 27 of H3 (H3K9me3 and H3K27me3) are markers of heterochromatin, indicating condensed, less susceptible, inactive chromatin, and are frequently associated with gene silencing [33,34]. Heterochromatin is essential for genomic stability [35]. A decrease in the global levels of the repressive marks H3K9me3 and H3K27me3 is linked to loss of heterochromatin [36]. The loss of heterochromatin occurs in the aging process and is associated with premature aging diseases [37,38], developmental disorders, and cancers [39,40,41]. A growing body of research evidence indicates the important role of histone methylation in disease onset and progression [42,43]. For example, decreased H3K27me3 is linked to ineffective DNA double-strand break repair [44]. The loss of H3K27me3 is a diagnostic and prognostic biomarker for cancers [45,46,47]. Increased active histone mark H3K4me3 and decreased repressive histone mark H3K9me3 have been found to be associated with a worse cancer prognosis [48].

Despite the increasing recognition of the importance of the early-life environment in health, it is a challenge to detect health issues later in life that are a result of early-life exposures. The incorporation of adverse health-effect-related epigenetic end points as earlier and more sensitive markers, especially in identifying the delayed effects, has great potential in understanding the exposure-related effects and offers better protection to the environment and public health [49]. Therefore, understanding the exposure effects during this critical time in association with epigenetic modifications is essential. Epigenetic change is one of the earliest molecular changes observed in response to environmental toxicant exposure and may be used as a predictive marker of sensitivity to environmental exposures [50,51,52] or disease prognosis [53,54,55].

Currently, the epidemiological evidence on the environmental epigenetic effect is focused on DNA methylation changes and is relatively limited in the areas of histone modifications [52,56]. Knowledge of prenatal PFAS exposures related to childhood epigenetic status is still lacking [57]. The aim of the present study was to explore how prenatal PFAS exposures interfered with H3K4me3, H3K9me3, and H3K27me3 among the 2-year-old children followed-up in a birth cohort study in Taiwan.

## 2. Materials and Methods

### 2.1. Study Participants and Questionnaire Data

The study participants were from the Taiwan Birth Panel Study (TBPS), a birth cohort study that recruited mother–infant pairs at birth at one regional hospital and two clinics in New Taipei City and one medical center in Taipei between April 2004 and January 2005. Trained interviewers used structured questionnaires to conduct in-person interviews within three days after delivery, and the participants were followed-up when the children were 2 years of age. The study participants were followed up when they turned 2 years old, as it has been suggested that the adverse environmental impacts on development are not apparent until the age of 2 years [58]. Structured prenatal and postnatal questionnaires were administered to collect the sociodemographic characteristics of the parents and potential exposure from the environment during pregnancy and childhood. Medical records were obtained to collect information on the gestational age, infant sex, and birth outcomes. The detailed information of this study has previously been described [59]. This study was approved by the Independent Ethics Committee of National Taiwan University Hospital. Informed consent was obtained from the mothers before giving birth to participate in this study and follow-up.

### 2.2. Sample Collection

Ten milliliters of umbilical cord blood at birth and peripheral blood from 2-year-old children were collected in ethylenediaminetetraacetic acid (EDTA) tubes. The blood specimens were separated into two tubes of whole blood and four tubes of plasma and buffy coat. The specimens remain frozen under −80 °C until the laboratory analyses were performed.

### 2.3. Total Histone Extraction and Histone Modification Analysis

The levels of global histone H3 at H3K4me3, H3K9me3, and H3K27me3 were measured in blood leukocytes from the 2-year-old children using Western blotting.

The histones were extracted from the buffy coat following the method by Cantone et al. [60]. The protein in each buffy coat sample was quantified by a bicinchoninic acid protein assay (Thermo Scientific™ Pierce™, Waltham, MA, USA) following the manufacturer’s protocol. An equal amount of protein (30 μg) was separated by 4–15% gradient SDS-PAGE gels at 200 V for 30 min and transferred to polyvinylidene fluoride membranes by using a sandwich electro-blotting system (Bio-Rad, Hercules, CA, USA) at 40 mA for 100 min on ice. Immunoblotting was performed by 2 h incubation with 5% PBST milk for membrane blocking; 1 h incubation using primary antibodies anti-H3 (1:5000; Millipore: 05-928), anti-H3K4me3 (1:20,000; Millipore: 04-745), anti-H3K9me3 (1:2000, Millipore: 07-442), H3K27me3 (1:20,000, Millipore: 07-449), and loading control anti-β-actin (1:10,000, Sigma-Aldrich: A5441); and 1 h incubation using secondary antibodies anti-rabbit (1:10,000, Thermo Fisher Scientific: 31460) and anti-mouse (1:80,000, Sigma-Aldrich: A9044). The gels were imaged using a UVP BioSpectrum Imaging System (Thermo Fisher Scientific, Waltham, MA, USA). The relative level of each band was measured by laser-scanning densitometry and analyzed by NIH Image J software 1.53 (Bethesda, MD, USA).

### 2.4. Exposure Measurements

The concentrations of the PFASs were measured in cord blood plasma samples. Details of the analyses have previously been described [61]. Briefly, the measurements of twelve PFASs, including PFOS, Me-PFOSA-AcOH, Et-PFOSA-AcOH, PFOSA, PFHxA, PFHpA, PFHxS, PFOA, PFNA, PFDeA, PFUA, and PFDoA, were performed using ultrahigh-performance liquid chromatography–tandem mass spectrometry (UHPLC/MS/MS) on the Waters ACQUITY UPLC system (Waters Corporation, Milford, MA, USA) with a Waters Quattro Premier XE triple quadrupole mass spectrometer. The laboratory experiments were conducted by personnel blinded to the characteristics of the study populations. The calibration curve of the standard solution showed high linearity (r^2^ ≥ 0.0996). The recoveries of PFASs determined by spiking were in the range of 85–104%, with relative standard deviations ranging from 0.02 to 6.37%. The intra- and inter-day calibration biases of all the analytes and concentrations were less than 7%, with relative standard deviations in the range of 0.02–8.22%. The limits of quantitation (LOQs), established based on a signal-to-noise ratio of ten, for PFOS, PFOA, PFNA, and PFUA were 0.22, 1.58, 0.84, and 3.1 ng/mL, respectively. PFASs with detection rates higher than 60% were included in the analyses, including PFOS, PFOA, perfluorononanoic acid (PFNA; C9), and perfluoroundecanoic acid (PFUA; C11), with detection rates of 98.9%, 85.1%, 67.6%, and 81.9%, respectively. The PFAS with levels less than their LOQs were allocated a value of one-half of the quantitation limits.

### 2.5. Statistical Analysis

Among the 486 mother–infant pairs recruited at birth, 130 of the study participants had blood samples collected at 2 years old. Since H3 cleavage may interfere with the measurement of histone modifications [62], study participants with H3 cleavage were excluded from the histone-methylation-related analyses. Global histone modification levels, including those of H3K4me3, H3K9me3, and H3K27me3, were calculated relative to the β-actin intensities. The PFAS measurements were natural log transformed because the distributions were right-skewed and then interquartile range (IQR) standardized. PFAS exposure has been found to be related to low birth weight, small for gestational age, and preterm birth based on the previous study of the study participants [13]. The histone methylation levels among the participants and the participants without adverse birth outcomes were evaluated to determine whether the adverse birth outcomes affected the histone methylation levels.

Selected characteristics were tested by *t*-tests for continuous variables and by χ^2^ or Fisher exact tests for categorized variables. The correlations between H3K4me3 and H3K9me3, H3K4me3 and H3K27me3, and H3K9me3 and H3K27me3 were calculated by Pearson’s correlation analysis. Univariable and multivariable regression analyses were applied to test the PFAS exposure’s effects on the histone methylation levels. The potential confounders considered to be adjusted in the multivariable regression models included parity, delivery method (vaginal delivery or cesarean section), maternal age, maternal BMI, parental education level, children’s sex, and age of the blood sample collected. The variables that changed the associations more than 10% were adjusted in the multivariable regression model to control for confounding effects.

All the tests were assumed to be two sided with a type I error of 0.05. The analyses were performed using SAS 9.4 (SAS Institute Inc., Cary, NC, USA) and R 3.5.2 (The R Project for Statistical Computing, Vienna, Austria).

## 3. Results

Of the 486 mother–infant pairs recruited at birth, 130 had blood samples collected at 2 years old. Twenty-six of them with H3 cleavage were excluded from the histone-modification-related analyses. The final study population included 104 participants. The characteristics of our study participants are shown in Table 1. The average age of the study participants when they had their blood samples collected was 2.2 (SD = 0.17; range = 2–2.67) years old. The average birth weight and gestational age were 3171 (SD = 498.8; range = 1024–4384) g and 38.5 (SD = 1.9; range = 29–41) weeks, respectively. Comparing with the total subjects in the TBPS, the study participants in the current analyses were 1.3 years older in maternal age, had higher parental education levels (98% of them had at least one of the parents graduate from college or university), and had a higher percentage of vaginal deliveries (70%). The birth outcomes were not significantly different between the followed-up participants and the non-followed-up ones. Compared with the total subjects in the TBPS, the study participants in the analyses had lower PFAS measurements (Table 2).

The correlation between H3K4me3 and H3K27me3 was higher (r = 0.63, *p* < 0.0001) than the correlations between H3K9me3 and H3K4me3 (r = 0.32, *p* = 0.005) and H3K9me3 and H3K27me3 (r = 0.33, *p* = 0.003). The associations between prenatal PFAS exposures and histone modification levels at 2 years old are presented in Table 3. The crude univariable models estimate the association without confounding adjustment. In the univariable models, we found that an IQR increase in PFUA exposure was associated with an increased H3K4me3 level by 2.02-fold (95% CI = (0.21, 3.83), *p* = 0.03). Each IQR increase in the PFOA and PFNA exposures was associated with a decreased H3k27me3 level by 2.25-fold (95% CI = (−4.11, −0.39), *p* = 0.01) and 2.13-fold (95% CI = (−4.05, −0.22), *p* = 0.03), respectively. In the multivariable models, after adjusting for parity, delivery method (vaginal delivery or cesarean section), maternal age, maternal BMI, parental education level, children sex, and age of the blood sample collected, an IQR increase in the natural log-transformed PFUA exposure was associated with an increased H3K4me3 level by 2.76-fold (95%CI = (0.79, 4.73), *p* = 0.007). Each IQR increase in PFOA and PFNA exposures was associated with a decreased H3K27me3 level by 2.35-fold (95%CI = (−4.29, −0.41), *p* = 0.01) and 2.01-fold (95%CI = (−4.00, −0.03), *p* = 0.04), respectively. We did not observe significant effects between PFOS and any of the histone methylation levels in either the univariable or the multivariable linear regression models. There were no significant differences in PFAS concentrations between participants with and without H3 cleavage.

## 4. Discussion

In this study, we examined the effects of prenatal exposures to PFASs on global histone modification levels among the 2-year-old children followed-up in a birth cohort study in Taiwan. Our results suggest that prenatal PFAS exposures were linked to an open chromatin configuration in 2-year-old children, including an increased level of active histone mark H3K4me3 with PFUA exposure and a decreased level of repressive histone mark H3K27me3 with PFOA and PFNA exposures.

To our knowledge, only a few birth cohort studies have reported the epigenetic changes in association with prenatal PFAS exposure, with epigenetic alterations focused on DNA methylation changes in cord blood and PFAS exposures focused on the effects of PFOA and PFOS [57]. In association with the global DNA methylation level in cord blood, Guerrero-Preston et al. reported that prenatal PFOA exposure was associated with decreased global DNA methylation level, a marker that leads to open chromatin and genomic instability [63,64], among the 30 infants in the Baltimore THREE (Tracking Health Related to Environmental Exposures) study [65], and our previous study reported that prenatal PFOS was associated with decreased Alu repeated elements, indicating decreased global DNA methylation among the 363 mother–infant pairs in the TBPS [66]. In association with the gene-specific and genome-wide DNA methylation levels in cord blood, several significant associations of prenatal PFOA and PFOS have been reported [67,68,69,70,71].

PFAS exposures have been found to affect the enzymes regulating DNA methylation and histone modifications [72,73,74]. DNA methylation and histone modifications are both essential during development [75]. The dysregulation of key regulatory histone modifications may affect the reprogramming that is required during development, such as bivalent chromatin maintenance marked by both H3K4me3 (activating) and H3K27me3 (repressive) histone modifications [76], and is frequently observed in developmental disorders [75,77] and cancer development and prognosis [78,79]. Exposure to endocrine-disrupting compounds early in life can modify epigenetic patterns and lead to a variety of later-onset diseases [51,80]. A study using undifferentiated SH-SY5Y neuroblastoma cells to mimic a developmental PFOA exposure effect found that PFOA exposure was linked to less-compact chromatin formation potentially through persistently reduced levels of repressive epigenetic marks, DNA methylation, and decreased levels of bivalent marks, H3K4me3 and H3K27me3, in neurons after the removal of PFOA and completion of neuron differentiation [81]. Studies of human breast epithelial cells found that PFOA exposure led to a persistent decrease in H3K9me2, a histone modification implicated in a closed chromatin structure, and a malignant transformation in the exposed cells and the unexposed daughter cells [82]. Rats exposed to PFOA had a decreased level of H3K9me3 and disturbed male reproductive endocrine in the testis [83]. Our results also showed decreased levels of H3K4me3, H3K9me3, and H3K27me3 with prenatal PFOA exposures among the followed-up 2-year-old children, but the association was only significant between H3K27me3 and PFOA. A decreased level of H3K27me3 has also been found in animals exposed to other endocrine-disrupting compounds [84,85,86]. Mice exposed to an organochlorine insecticide, chlordecone (CD), during embryonic development had increased H3K4me3 and decreased H3K27me3 levels in the prostate and an increased prostatic intraepithelial neoplasia phenotype [86]. Studies in rats found that neonatal exposure to the xenoestrogen diethylstilbestrol (DES) and the environmental estrogen genistein reduced H3K27me3 levels [83,84] and increased uterine fibroids and tumor incidence [87]. An in vivo study using fly models demonstrated that PFOA exposure significantly affected the methyltransferase activity of the enhancer of zeste homolog 2 (EZH2), a H3K27 methyltransferase, and metastasis [88]. These xenoestrogens have been shown to reduce EZH2 and lead to reduced H3K27me3 levels [84]. Further studies are required to provide an insight into the mechanisms of the observed alterations in histone modifications in association with PFAS exposures.

PFASs are known to have elimination half-lives in years and are widely distributed in the environment, with the half-lives and persistence increasing with the carbon chain length [8,89]. Many countries have made an effort to eliminate or limit the use of PFOA and PFOS in industrial processes. The concentrations of PFOA and PFOS in human blood have decreased in North America and Europe after 2000, while the concentrations of other PFASs, including PFNA and PFUA, have increased [90,91,92]. The PFOA and PFOS levels according to our study were found to be lower than those in Canada [93], Denmark [12,94], and Norway [95] but higher than those in Japan [68] and the United States [11,65]. A relatively high PFUA level was found in our study population and has also been reported in populations with a high seafood consumption in Asia [96]. The health effects of these PFASs are still of concern. For example, prenatal PFUA and PFNA exposure has been found to affect performance and verbal intelligence quotient (IQ) in children at 5 and 8 years old [97] and to cause thyroid function impairment in children and adolescents [14,98]. In utero exposure that alters histone methylation levels may contribute to an environment that increases the risk of adverse health outcomes later in life. However, at present, health predictions cannot be made based solely on histone modification levels.

In summary, our study provides additional evidence that prenatal PFAS exposure disrupts the epigenetic machinery that regulates histone modifications in children. There are only a few birth cohorts that have PFAS measurements available; our findings contribute to understanding the PFAS-exposure-related effects. The potential limitations of this study include, first, that the histone modification levels were measured in blood. The results may not be applicable to the other tissues or organs, though blood is widely used to test for exposure or disease status. Our findings may only be able to represent an early stage of the systemic effect from exposure. Second, even though we adjusted for several potential confounders based on the literature, potential confounding effects such as the effects of exposure to heavy metals or insecticide during the life course may remain. Third, the lack of postnatal PFAS exposure measurements is another limitation. Postnatal PFAS exposure, such as that occurring during lactation, contributes to part of the overall perinatal PFAS exposure and should be carefully considered [99]. Fourth, the sample size of this study limits our ability to test for interaction effects such as the differential exposure effect by sex. Fifth, only 104 of 486 total participants in the TBPS were included in the analyses; selection bias is a potential concern. The included participants were older in maternal age, had higher parental education levels, a higher percentage of vaginal deliveries, and lower PFAS measurements. We adjusted for several potential confounders including maternal age, parental education levels, and delivery method and found the results remained significant. Further research is required to confirm the findings.

## 5. Conclusions

The results from this study suggest that prenatal exposure to PFASs is associated with global histone methylation changes in 2-year-old children.

## Figures and Tables

**Table 1:** Baseline characteristics of total and included study participants for histone modification analysis.

Characteristics ^a^	Total (N = 486)	Excluded (N = 382)	Included (N = 104)
Maternal age at delivery (years) *	30.8 (4.7; 18–45)	30.5 (4.7; 18–45)	32.1 (4.4; 19–42)
Maternal BMI (kg/m^2^)	20.9 (3.1; 14.2–37.4)	20.9 (3.2; 14.2–37.4)	20.7 (2.5; 15.8–28.8)
Parental education level ^b^			
Not senior high school graduated (%)	3 (0.6)	3 (0.8)	0 (0)
Senior high school graduated (%)	24 (4.3)	19 (5.0)	2 (1.9)
Four-year college/university and above (%)	485 (95.1)	359 (94.2)	102 (98.1)
Children			
Boys (%)	246 (50.6)	188 (49.2)	58 (55.8)
Delivery method, vaginal (%) *	292 (60.1)	219 (57.3)	73 (70.2)
Birth outcomes			
Gestational age (weeks)	38.5 (1.7; 27–41)	38.5 (1.7; 27–41)	38.5 (1.9; 29–41)
Preterm birth (<37 weeks) (%)	42 (8.6)	35 (9.2)	7 (6.7)
Birth weight (g)	3157.9 (476.6; 772–5100)	3154.2 (470.4; 772–5100)	3171 (498.8; 1024–4384)
Low birth weight (<2500 g) (%)	28 (5.8)	23 (6.1)	5 (4.8)
Small for gestational age (%)	32 (6.6)	26 (6.8)	6 (5.8)

^a^ All characteristics are expressed as mean (SD; range) or percentage. ^b^ Highest education level of either parent. * *p* < 0.05.

**Table 2:** Distribution of PFAS exposure measurements in cord blood between the total and included study subjects.

Exposure ^a^ (ng/mL)	Detection Limit (ng/mL)		Mean	(SD)	GM	(GSD)	Median
PFOS	0.066	Total	7.66	(7.34)	5.97	(1.95)	5.67
		Included	6.66	(4.23)	5.68	(1.74)	5.49
		Excluded	7.93	(7.96)	6.05	(2.01)	5.68
PFOA	1.23	Total	2.59	(2.40)	1.84	(2.24)	1.86
		Included	2.56	(2.45)	1.77	(2.32)	1.74
		Excluded	2.6	(2.39)	1.85	(2.24)	1.89
PFNA	0.67	Total	6.31	(8.39)	2.38	(4.70)	3.00
		Included	5.89	(7.53)	2.21	(4.74)	2.82
		Excluded	6.42	(8.61)	2.43	(4.69)	3.02
PFUA *	2.4	Total	16.89	(15.9)	10.12	(3.11)	13.50
		Included	14.26	(14.2)	7.93	(3.32)	10.29
		Excluded	17.59	(16.3)	10.73	(3.05)	14.46

Abbreviations: GM, geometric mean; GSD, geometric standard deviation; PFASs, perfluoroalkyl substances; PFNA, perfluorononanoic acid; PFOA, perfluorooctanoic acid; PFOS, perfluorooctyl sulfonate; PFUA, perfluoroundecanoic acid. ^a^ Concentration values below the limits of quantitation were set to be 1/2 the limit of quantitation. * *p* < 0.05.

**Table 3:** The associations between PFAS exposures and global histone methylation levels.

Global Histone Modifications (Relative%)	Exposure ^a^	Crude Linear Regression Model			Multiple Linear Regression Model ^b^		
		*β*	(95% CI)	*p*-Value	*β*	(95% CI)	*p*-Value
H3K4me3	PFOS	−1.29	(−3.42, 0.84)	0.23	−1.36	(−3.90, 1.17)	0.29
	PFOA	−2.42	(−5.07, 0.23)	0.07	−2.38	(−5.32, 0.57)	0.11
	PFNA	−1.11	(−3.87, 1.65)	0.42	−0.82	(−3.86, 2.21)	0.59
	PFUA	**2.02**	**(0.21, 3.83)**	**0.03**	**2.76**	**(0.79, 4.73)**	**0.007**
H3K9me3	PFOS	−0.55	(−1.35, 0.26)	0.18	−0.52	(−1.38, 0.33)	0.22
	PFOA	−0.05	(−1.09, 0.98)	0.92	0.14	(−0.88, 1.16)	0.78
	PFNA	−0.11	(−1.17, 0.94)	0.83	0.19	(−0.84, 1.21)	0.71
	PFUA	−0.62	(−1.31, 0.08)	0.08	−0.22	(−0.93, 0.49)	0.53
H3K27me3	PFOS	−0.52	(−2.05, 1.02)	0.50	−0.42	(−2.15, 1.31)	0.63
	PFOA	**−2.25**	**(−4.11, −0.39)**	**0.01**	**−2.35**	**(−4.29, −0.41)**	**0.01**
	PFNA	**−2.13**	**(−4.05, −0.22)**	**0.03**	**−2.01**	**(−4.00, −0.03)**	**0.04**
	PFUA	0.18	(−1.16, 1.51)	0.79	0.87	(−0.53, 2.28)	0.22

^a^ Exposure levels are natural log transformed and interquartile range (IQR) standardized. ^b^ Multivariable regression model adjusted for parity, delivery method (vaginal delivery or cesarean section), maternal age, maternal BMI, parental education level, children sex, and age of the blood sample collected.

## Data Availability

The data are unavailable due to privacy or ethical restrictions.
